# Elicitation of Health-related Quality-of-life Concepts Associated with Overactive Bladder: A Qualitative Study

**DOI:** 10.36469/9816

**Published:** 2016-08-09

**Authors:** Natalia Hawken, Zalmai Hakimi, Samuel Aballéa, Jameel Nazir, Isaac A. O. Odeyemi, Mondher Toumi

**Affiliations:** 1 Creativ-Ceutical SARL, Paris, France; 2 Astellas Pharma Europe B.V., Astellas Medical Affairs, Global, HEOR, Leiden, The Netherlands; 3 Astellas Pharma Europe Ltd., Astellas Medical Affairs, EMEA, HEOR, Chertsey, UK; 4 University Claude-Bernard Lyon-I, Lyon, France

**Keywords:** patient-centered outcomes research, urinary incontinence, qualitative research, quality of life, overactive, urinary bladder

## Abstract

**Background:** Overactive bladder (OAB) is a symptom-defined disorder. A range of instruments are available for assessing OAB symptom bother, urinary urgency and the effects of symptoms on health-related quality of life (HRQoL), but few have been specifically designed and validated for this condition. HRQoL instruments should capture the concepts that are most relevant to patients. To our knowledge, there is no existing published conceptual framework for OAB.

**Objectives:** We performed a qualitative study to explore the impact of symptoms of OAB on affected patients and to develop a conceptual framework for OAB.

**Methods:** Patients diagnosed with OAB living in the United Kingdom were interviewed on the telephone by a trained psychologist using an interview discussion guide. Interview transcripts were analyzed thematically by two psychologists. Data collection and analysis was completed when data saturation, i.e. when little or no new information was obtained, was achieved.

**Results:** A total of 30 patients were interviewed. Fifteen patients (50%) had urge incontinence (i.e. OAB-wet). Interview data showed that OAB affected role functioning, sleep quality, social functioning, and emotional/mental functioning. In addition, patients often adopted non-medical coping strategies to manage their symptoms (e.g. planning activities). Factors which affected more than 50% of patients were going for a short walk, waking up at night, travelling/holidays, socializing/going out, embarrassment/shame, need to plan activities, and restriction of places visited. More patients with OAB-wet reported impairment of social and emotional/mental functioning than patients with OAB-dry. A conceptual framework for adults with OAB depicting the relationships between OAB concepts (or outcomes) was developed.

**Conclusions:** OAB has a profound effect on patient HRQoL and negatively affects a broad range of functions, including daily and work activities, leisure and social activities, psychological well-being, and sleep capacity. The conceptual framework emerging from this study supports the utilization of existing disease-specific HRQoL instruments, but identifies that work-related effects, which are relevant for OAB patients, are missing from currently available measures.

## BACKGROUND

Overactive bladder (OAB) is a symptom-defined disorder. According to the International Continence Society, it is characterized by urinary urgency, which is usually accompanied by urinary frequency and nocturia.[1] These symptoms may or may not be accompanied by urge incontinence, i.e. OAB-wet and OAB-dry, respectively. OAB is a common and debilitating condition. Large community population-based surveys suggest that between 11% and 17% of adults have symptoms[2–4] and an estimated 500 million people suffer from OAB worldwide (modelled data for 2013).[5] The prevalence of OAB increases with age2 and symptoms are typically more prevalent in women than in men.[2,5] Symptoms of OAB compromise health-related quality of life (HRQoL) and can have a negative effect on social, psychological, occupational, domestic, physical, emotional and sexual areas of life.[6]

Objective measures of treatment efficacy (e.g. micturition frequency and incontinence frequency) are almost always used in clinical trials, but the symptom-based nature of OAB means that patient input should also be considered and used to evaluate treatment response. A growing body of evidence further suggests that objective measures are often poorly representative or correlated with patient-reported outcomes (PRO).[7] Currently a range of instruments are available for assessing symptom bother, urinary urgency and the effects of OAB symptoms on HRQoL, but only a few (e.g. Overactive Bladder Questionnaire [OAB-q[8]) have been designed and validated specifically for this condition.[9]

PRO instruments should capture the HRQoL concepts, or outcomes, that are most important and relevant to patients.[10] Concept elicitation through qualitative research is a key step to understanding a patient's perspective of their condition. Good research practice guidelines for eliciting concepts recommend developing a conceptual framework based on qualitative studies, a conceptual framework being "a diagram depicting relationships between the overarching concept, hypothesized domains or subconcepts, and candidate item content."[10] There is currently limited formal qualitative research relating to OAB and, to our knowledge, there is no existing published conceptual framework for OAB. We performed a qualitative study based on grounded theory to explore the impact of OAB symptoms on affected patients, particularly their self-esteem, social and personal relationships, and to identify key concepts of relevance for OAB. This information was then used to build a conceptual framework depicting the relationships between each of the OAB concepts.

## MATERIALS AND METHODS

The study consisted of two separate stages: (i) a literature review; and (ii) patient concept elicitation interviews. It was conducted in accordance with the codes of ethics and conduct defined by the American Psychology Association,[11] and also with the recommendations of the International Society for Pharmacoeconomics and Outcomes Research (ISPOR) PRO Good Practices Task Force.[10] Informed consent was obtained from all individual participants included in the study; no identifiable information about the patients was gathered. Ethical approval for this study was granted by the Solihull NRES Committee (West Midlands Health Research Authority) – 15/WM/0296.

### Literature Review

A literature review was performed to identify existing qualitative evidence regarding PRO related to OAB symptoms and management. The findings of the literature review were used to form the basis of a theoretical conceptual framework for OAB.

The search was performed using the EMBASE database (2003–2013) to identify qualitative studies reporting patient interviews conducted to gauge the impact of OAB. Studies were also required to meet the following criteria:

- English language
- Adults (men or women) with OAB
- Any sample size
- Any country
- Reported results on quality of life, well-being, functioning, or satisfaction with life or treatment
- Reviews, case studies and letters were excluded.

In addition, the Cystitis and Overactive Bladder Foundation website[12] was searched for relevant HRQoL information reported by OAB patients.

### Patient Interviews

To better understand the important concepts of OAB from a patient's point of view, interviews with individual patients were conducted using an interview guide.

*Interview Discussion Guide:* Telephone interviews were conducted with two urologists practicing in Europe to identify key HRQoL concepts of relevance to patients with OAB. This was achieved by discussing patients' experience of the disease and treatment, and existing evidence regarding PRO for patients with OAB. The findings from these interviews were used to develop an interview guide for OAB patients (Supplementary Appendix A).

*Patients:* Patients were required to meet the following inclusion criteria: diagnosis of OAB (OAB-wet or OAB-dry) at least 6 months before the interview; English speaking; and living in the UK. Incontinent or OAB-wet patients were included only if they were suffering from urge incontinence, and were at least somewhat bothered (according to a 5-point Likert scale – not at all; a little; somewhat; much; a great deal) by the following: (i) urine loss associated with a strong desire to urinate; and (ii) a sudden urge or desire to urinate that makes the patient want to stop what they are doing and rush to the bathroom but they do not get there in time (i.e. urinary leakage or wet pads). Patients with the following conditions were excluded: stress incontinence; multiple sclerosis; patients with a history of drug abuse or dependence within the past year, excluding nicotine and caffeine; patients with evidence of a progressive central nervous system (CNS) disease (e.g. CNS lupus, tumors, multiple sclerosis, Alzheimer's disease), lesion, or encephalopathy; and patients with significant psychiatric or neuropsychiatric disorders (e.g. severe depression, bipolar disorder or schizophrenia spectrum disorder, history of suicide attempt, or recent history of suicidal ideation).

Patients were recruited through general practitioners (GPs). If patients met the inclusion criteria, they were provided with information about the study over the phone. They were then sent a printed leaflet detailed with information regarding the purpose and format of the interview and given a few days' reflection time before another phone call to see if they wanted to participate. An in-depth interview by telephone was arranged with patients who agreed to participate. Before the start of each interview, patients were reminded about the purpose and format of the interview, their right to withdraw from the interview without explanation and asked to confirm their willingness to participate; these steps, including verbal consent, were recorded in the transcripts.

*Interviews:* A trained psychologist (NH; 10 years' experience) conducted semi-structured telephone interviews with individual patients using the interview discussion guide (Supplementary Appendix A). The interviews were conducted between 6 May 2013 and 17 July 2013, and each lasted for approximately 1 hour (maximum 1.5 hours). The interviews provided an unobtrusive way of gaining insight and allowed participants to openly express their thoughts and feelings, while allowing the interviewer to keep the interview focused on the key factors which affect HRQoL. Specific discussion topics included:

- History of OAB, i.e. when the disease appeared, how the patient was diagnosed and what symptoms were experienced.
- Impact of OAB, i.e. what it is like to suffer from OAB and how the patient's life had been affected by OAB (e.g. relationships, activities, self-image, sleep).
- Important areas of impact, i.e. what are the most important areas of impact, how they could be improved, and what level of improvement would have a positive impact on HRQoL.

All interviews were audiotaped and transcribed verbatim. A quality check of the transcripts was performed (i.e. checked for accuracy against the audio tapes).

*Data Analysis:* Thematic analysis was used to identify key areas of impact of OAB from the interview transcripts, and facilitated by Atlas.ti software. Thematic analysis potentially provides a rich and detailed, yet complex, account of data.[13] Thematic analysis of qualitative research is a well-documented approach in social sciences and has been used successfully to explore dimensions of quality of life and to support data derived from quantitative self-report measures by providing added depth.[14] The transcript text was read several times and portions that related to broad categories were marked. Subthemes within those thematic categories were coded. Statements that provided links between themes were also identified. Saturation tables, organized by concept code, were developed to document the elicitation of information in successive interviews. Data collection and analysis was completed when data saturation was achieved (i.e. new information produced little or no change to the code book). Concepts reported by patients were presented for all patients, as well as for patients with OAB-wet and OAB-dry.

## RESULTS

### Literature Review

Three qualitative studies, which used individual patient interviews or patient focus groups to understand the effects of OAB on quality of life, well-being, functioning or satisfaction with life, were identified from the literature search.[15–17] The results of the studies show that:

- OAB is a debilitating disease with a serious impact on quality of life.
- OAB affects patients' sexual health, reduces sexual desire and the ability to achieve orgasm.
- OAB affects HRQoL, self-esteem and relationships.
- OAB without incontinence causes anxiety, fear of incontinence, a sense of depression and hopelessness, all of which are worse for those with incontinence.
- As a chronic condition, OAB also affects lifestyle (self-limited, isolated, and sedentary), which in turn affects self-esteem and future prospects.
- The longer term implications of living with OAB manifest as a sense of despair and loss.

A summary of the symptom and impact concepts identified from the studies is shown in Supplementary Table 1. From this summary, an initial theoretical conceptual framework was developed to describe the relationship between the symptoms of OAB (i.e. frequency, urgency, incontinence and nocturia) and their impact on HRQoL in the areas of daily living, social functioning, and sexual and mental health (Supplementary Figure 1).

### Patient Interviews

A total of 30 patients were interviewed. The demographics and symptoms experienced by patients at the time of interview are shown in Table 1. Patients were diagnosed with OAB between 2007 and 2013, at least 6 months prior to interview. All patients reported that they needed to void at least 8 times per day (range 8–16). All patients had urgency and frequency of urination; 50% of patients had urge incontinence and were classified as patients with OAB-wet.

**Table 1.** Patient Demographics and Symptoms

| | OAB-wet Patients (n=15) | OAB-dry Patients (n=15) | Total (n=30) |
|---|---|---|---|
| **Age** | | | |
| Median Years (Range) | 63 (26–83) | 58 (37–79) | 61 (26–83) |
| >65 Years, n (%) | 10 (15) | 6 (15) | 16 (53) |
| **Sex, n (%)** | | | |
| Male | 4 (27) | 3 (20) | 7 (23) |
| Female | 11 (73) | 12 (80) | 23 (77) |
| **No. Voids per Day** | | | |
| Range | 8–16 | 8–10 | 8–16 |
| **Symptoms, n (%)** | | | |
| Urge Incontinence | 15 (100) | 0 (0) | 15 (50) |
| Urgency of Urination | 15 (100) | 15 (100) | 30 (100) |
| Frequency of Urination | 15 (100) | 15 (100) | 30 (100) |
| Nocturia | 15 (100) | 13 (87) | 28 (93) |

The data from patient interviews show that OAB affects several key aspects of patient HRQoL, i.e. role functioning (e.g. daily and work activities), sleep quality, social functioning (e.g. leisure and social activities), and emotional/mental functioning (e.g. embarrassment, fears and worry) which forced patients to adopt coping strategies to manage their OAB symptoms. Illustrative quotes from the patient interviews for each of the key concepts are presented in Table 2.

**Table 2.** Concepts and Illustrative Quotes Pertaining to Those Concepts

| Concept | Quotes |
|---|---|
| **Role Functioning** | |
| Role at Work | "Well, it basically gutted me. I couldn't go to work and constantly be running to the loo. A lot of my work was outside work, you see. [...] I gave my job up." (Female, 39 years) |
| Role as a Caregiver or as Family Member | "It has become more difficult, because picking my daughter up, I have to be careful how I pick her up." (Female, 34 years) |
| | "Well, what is bothering me the most really is my quality time with my grandchildren, because at the end of the day, I've got three grandchildren and I enjoy spending time with them, you know." (Male, 63 years) |

**Table 2.** Concepts and Illustrative Quotes Pertaining to Those Concepts - continued

| Concept | Quotes |
|---|---|
| **Sleep and Daytime Functioning** | |
| Waking Up at Night | "And also, I wake up once maybe twice of the night to go to the toilet, so that disrupts my sleep." (Female, 59 years)<br>"And then I'll probably get up once in the night to go to the toilet." (Female, 66 years)<br>"It always takes me a long time to actually to get to sleep the first time, when I am going, […] I will be up three times, […]." (Female, 70 years)<br>"And once disturbed, getting back to sleep is a nightmare. Literally. I can't get back to sleep very easily. It disturbs my sleep time. And then it's quite hard to get back to sleep." (Female, 61 years) |
| Impaired Daytime Functioning | "Oh, yes, it makes you tired, yeah. It makes you tired because you're not getting your proper rest through the night." (Female, 72 years)<br>Interviewer: "Do you ever feel tired in the morning because you had to get up at night?"<br>Patient: "Yeah, I do feel tired most mornings." (Female, 34 years) |
| Daytime Sleeping | "Yeah, the next day I feel sleepy and tired. And, during the day, sometimes, I know to take 15 minutes off - Okay." (Male, 72 years)<br>"Yeah, because I have to stay up until the afternoon and I can't keep on having a nap in the afternoon because I'm so tired, […], in the afternoon, so you have to have a power out for half an hour or so." (Male, 34 years)<br>"If I've had a really bad night, I might have spent the afternoon sit in a chair and fall asleep for a half an hour or so. It just sort of made me sort of sleep in the afternoon a bit. I mean it, some days it does, but I don't do that, but if I'm home here and I am not at work, I would sort of, more likely than not, if I didn't fall asleep for forty minutes in the afternoon, if I'd had a meal at tea time, I probably would fall asleep a bit then." (Female, 72 years) |
| Irritability, Anger, Annoyance | "And of course, when I'm not sleeping enough, it makes me drowsy, it makes me annoyed sometimes, you understand what I mean?" (Male, 72 years)<br>"It was annoying because I was not having a full night sleep." (Male, 63 years)<br>"I felt irritable, and angry with myself, because I was having disturbed nights." (Male, 48 years)<br>"Getting up at night, no sleep, extremely tired, extremely irritable." (Female, 75 years)<br>"Yeah, because I have to stay up until the afternoon and I can't keep on having a nap in the afternoon because I'm so tired, and you feel so drained and depressed and drowsy." (Male, 63 years)<br>"I suppose [inaudible] as I said, you've just got to get on with it. I do get a lot of headaches, but that's obviously the stress or maybe just because I'm tired because I'm never, I never get a full night solid sleep, I always have broken sleep, you know, so…" (Female, 64 years) |
| Impaired Concentration | Interviewer: "…Do you get tired or like get difficulties to concentrate these kind of things?"<br>Patient: "That's one thing I do have sometimes a little loss of concentration […] and that was a lack of concentration and that's definitely down to lack of sleep […] so yeah I do notice lack of sleep gives you lack of concentration." (Female, 64 years) |
| Impaired Work Performance | "I'm an Auditor in the hotel, so I'm dealing with obviously the money the financial side in the evenings so obviously I can't mess up on those […] so like last night for example I made a mistake and I corrected it straightaway." (Male, 39 years) |
| Impaired Social Life | "I mean, I don't go out early in the morning, I'm too tired to get up, you know, I have to come around because I have not got a very good night's sleep, so I don't go out really, unless it's really I have to. And then, it's an effort, it's because, you know, I'm tired. Quite tired." (Female, 72 years) |

| Concept | Quotes |
|---|---|
| **Sleep and Daytime Functioning (continued)** | |
| Impaired Social Life (continued) | "It's very rare now that I got to get up two, three, four o'clock in the morning that means if I'd, [coughs] it means I could not get back to sleep so I have been awake from 2 o'clock to morning throughout the whole day, until I go to bed at night, sometimes at 6 o'clock at night. So, the social life was basically nothing. Getting up at night, no sleep, extremely tired, extremely irritable." (Female, 72 years) |
| Disturbing Partner | "It was annoying because I was not having a full night sleep. I suppose in a way, I was disturbing my husband because I was getting in and out of bed, because the bathroom, as I said, is downstairs." (Female, 64 years) |
| **Social Functioning** | |
| Avoiding Socializing/ Going Out | "And I am getting to the stage where I am frightened to leave the house." (Female, 61 years)<br><br>"You get on with it, now I'm on my own. I haven't seen as much with my friends so often, and stuff like that, you know." (Female, 34 years) |
| **Emotional/Mental Functioning** | |
| Worry | "You know, you're always worried in case it smells, or something." (Female, 66 years)<br><br>"Because if I go to the toilet I can wipe myself down just in case. I do wear panty liners, and I carry them with me so I can change myself. If my daughter said mum you smell, I think I'd be so embarrassed and so upset." (Female, 39 years)<br><br>"But it's embarrassing if it happens in somebody's house, sometimes, you have to go, you know, every 15 minutes and, what were you drinking all morning?" (Female, 63 years)<br><br>"I suppose they are not very accessible, but I suppose I just don't like people seeing me go to the toilet, you know, all the time, sort of thing. I try and keep it secret." (Female, 75 years) |
| Anxiety | "Well, let me tell you it's ongoing, it's a bit different. I'm always anxious all the time. I've got to make sure I've got my pads and there's a toilet somewhere, where I'm going. I'm anxious all the time." (Female, 75 years)<br><br>"I get a bit stressed about not making it to the toilet, but then I learned to accept it. If I have an accident, I have an accident." (Female, 70 years)<br><br>"It makes me embarrassed, because I go to a few work conferences and things like that, and I always have to get up and, you know leave and come back in, and obviously people see me going quite often, and I find it sort of embarrassing." (Female, 75 years) |
| Embarrassment/ Shame | "And it's very embarrassing because if you go on holiday, as I said I'm a widow." (Female, 65 years)<br><br>"I think it's just the social aspect and feeling embarrassed about it. Because people do notice how many times you go to the loo." (Female, 70 years)<br><br>"Because if I go to the toilet I can wipe myself down just in case. I do wear panty liners, and I carry them with me so I can change myself. If my daughter said mum you smell, I think I'd be so embarrassed and so upset." (Female, 39 years)<br><br>"And also I've had to start wearing incontinent pads at night which I do find very embarrassing." (Female, 70 years) |
| Anger | "I was feeling angry and tired, because I haven't had a good night sleep." (Male, 63 years) |
| **Sexual Impact** | |
| | "Well, it's quite difficult with me and my partner especially. You know, it's hard that we can't sit and watch a film or have a cuddle, if you know what I mean." (Female, 26 years) |
| **Coping Strategies** | |
| Wearing Pads | "I always use the mini pads, yes." (Female, 70 years) |

| Concept | Quotes |
|---|---|
| **Coping Strategies (continued)** | |
| Wearing Pads (continued) | "And also I've had to start wearing incontinent pads at night which I do find very embarrassing." (Female, 70 years) |
| Need to Have Extra Clothing | "Because if I go to the toilet I can wipe myself down just in case. I do wear panty liners, and I carry them with me so I can change myself." (Female, 39 years) |
| Restriction of Drink Types (Tea/Coffee/Soda/Juice/Alcohol) | "He told me avoid, uh, tea and caffeine, coffee, caffeine, [indiscernible], juice, stuff like that, uh, I think, because that irritates the bladder." (Male, 72 years) |
| | "Um… no, I wouldn't, I think rather than to have two cups of tea before I went out, I'd just have the one." (Female, 74 years) |
| | "But if I had a cup of tea, or a cup of coffee, I can go four times an hour. So I do try and not, you know, drink any caffeinated products, and just take a sip of water here and there." (Female, 70 years) |
| Drinking Only Decaffeinated Tea and Coffee | "I only drink decaffeinated tea and coffee." (Female, 70 years) |

*Role Functioning:* Several patients reported that their bladder condition interfered with their ability to function normally at work. A few patients reported that their bladder condition was getting in the way of their ability to fulfill their role as a caregiver or family member.

*Sleep and Daytime Functioning:* Most patients (93%) presented with nocturia. Sleep disturbances (such as difficulty maintaining sleep, early morning awakening) were identified as a significant problem for patients, as they adversely affected daytime functioning, caused fatigue and impaired concentration. Irritability, anger, annoyance, stress and depression were reported as a consequence of disturbed sleep. Consequently, OAB patients reported that their work performance and general daily functioning declined. Lack of sleep quality and quantity led patients to compensate by having morning naps. Patients also reported going to bed earlier than others (e.g. family members, friends) and that they had "no social life". One patient reported that waking up several times during the night was likely to disturb her partner.

*Social Functioning:* People avoided leaving their house or restricted their activities outside of home because of OAB symptoms. Incontinence, in particular, had a devastating impact on self-confidence and was the major source of embarrassment. Patients also preferred to stay at home so as not to have to worry about using other people's toilets or to avoid the distress of looking for a public toilet. One patient reported evading family holidays to avoid disturbing their travel companions. Inevitably, this led to feelings of isolation. The impact of OAB on sexual function was not well captured in our sample as only 3 of the 30 participants discussed it.

*Emotional and Mental Functioning:* Worry was an important issue for patients with OAB. They wanted to avoid others noticing their condition and feared smelling of urine. Fear of incontinence was also a source of anxiety for many OAB patients, and the potential embarrassment associated with urinary leakage in public. Most women experienced embarrassment and/or shame because of their condition. Patients also reported worrying about the fact that people noticed their frequent use of the toilet. Not knowing the location of toilets was also a source of worry. Tiredness also impacted on mood, and anger was common.

*Coping:* Patients reported using several different strategies to manage their symptoms, including wearing pads, carrying extra clothing, restricting certain drinks (i.e. tea, coffee, alcohol) and drinking decaffeinated tea and coffee.

A summary of the data from the saturation tables is shown in Table 3. Seven factors affected more than 50% of patients: going for a short walk; waking up at night; travelling and holidays; socializing or going out; embarrassment/shame; need to plan activities; and restriction of places visited. Most patients (n=27/30) did not discuss the impact of OAB on sexual function.

When comparing patients with OAB-wet vs. OAB-dry (Table 3), more patients with OAB-wet reported concepts relating to social and emotional/mental functioning or well-being, particularly satisfaction with life (93% of patients with OAB-wet vs. 0% of patients with OAB-dry), embarrassment/shame (93% vs. 67%), fear of clothing smelling of urine (40% vs. 13%) and socializing/going out (100% vs. 47%).

Patients were also asked to rate the most bothersome symptom of OAB. All patients with OAB-wet rated urge incontinence as the most bothersome symptom, whereas frequency (10 patients) and urgency (5 patients) were identified as the most bothersome symptoms by OAB-dry patients.

Based on these data, a conceptual framework for adults with OAB was developed and is shown in Figure 1.

**Table 3.** Factors Affecting Quality of Life in Patients with OAB (Reported by >5% of Patients)

| Concept | No. Patients (%) | | |
|---|---|---|---|
| | **OAB-wet (n=15)** | **OAB-dry (n=15)** | **Total (n=30)** |
| **Role Functioning** | | | |
| Shopping | 3 (20) | 1 (7) | 4 (13) |
| Impact on Work | 3 (20) | 4 (27) | 7 (23) |
| **Sleep** | | | |
| Waking Up in the Night | 14 (93) | 15 (100) | 29 (97) |
| Difficulties Falling Back to Sleep | 3 (20) | 5 (33) | 8 (27) |
| **Social Functioning** | | | |
| Going for a Walk | 12 (80) | 14 (93) | 26 (87) |
| Travelling and Holidays | 8 (53) | 9 (60) | 17 (57) |
| Socializing/Going Out | 15 (100) | 7 (47) | 22 (73) |
| Sport* | 3 (20) | 1 (7) | 4 (13) |
| Intimate Relationships | 2 (13) | 1 (7) | 3 (10) |
| Family Activities | 2 (14) | 2 (14) | 4 (14) |
| **Emotional/Mental Functioning** | | | |
| Embarrassment/Shame | 14 (93) | 10 (67) | 24 (80) |
| Satisfaction with Life | 14 (93) | 0 (0) | 14 (47) |
| Perception of Others | 5 (33) | 4 (27) | 9 (30) |
| Fear of Clothing Smelling of Urine | 6 (40) | 2 (13) | 8 (27) |
| Annoyance/Frustration/Irritability | 6 (40) | 8 (53) | 14 (47) |
| Anxiety/Stress | 4 (27) | 3 (20) | 7 (23) |
| Worry about Having Leakages | 5 (33) | 3 (20) | 8 (27) |
| Worry that Others will Notice Frequency of Going to the Toilet | 3 (20) | 2 (13) | 5 (17) |

*Running, swimming, cycling, golf, ice-skating
Note: Factors affecting more than 50% of patients are underlined.

| Concept | No. Patients (%) | | |
|---|---|---|---|
| | OAB-wet (n=15) | OAB-dry (n=15) | Total (n=30) |
| **Emotional/Mental Functioning (continued)** | | | |
| Worry about Reaching Toilet in Time | 5 (33) | 2 (13) | 7 (23) |
| Fear of Leaving Home/Finding Toilets when Outside | 5 (33) | 4 (27) | 9 (30) |
| Fatigue | 3 (20) | 4 (27) | 7 (23) |
| Depression | 4 (27) | 2 (13) | 6 (20) |
| Self-perception | 4 (27) | 2 (13) | 6 (20) |
| Physical Discomfort | 3 (20) | 2 (13) | 5 (17) |
| Motivation to do Things | 2 (13) | 3 (20) | 5 (17) |
| Anger | 0 (0) | 3 (20) | 3 (10) |
| Self-confidence | 2 (13) | 0 (0) | 2 (7) |
| Isolation | 1 (7) | 1 (7) | 2 (7) |
| **Coping Strategies** | | | |
| Need to Plan Activities | 10 (67) | 9 (60) | 19 (63) |
| Restriction of Places Visited | 6 (40) | 10 (67) | 16 (53) |
| Restriction of Fluid Intake | 5 (33) | 10 (67) | 15 (50) |
| Restriction of Drink Types (Tea, Coffee, Soda, Juice, Alcohol) | 5 (33) | 4 (27) | 9 (30) |
| Wearing Pads | 5 (33) | 2 (13) | 7 (23) |
| Need to have Extra Clothes | 1 (7) | 1 (7) | 2 (7) |

Note: Factors affecting more than 50% of patients are underlined OAB: overactive bladder

**Figure 1.** Conceptual Framework of Health-related Quality-of-life Concepts Associated with Overactive Bladder, Based on Patient Interviews

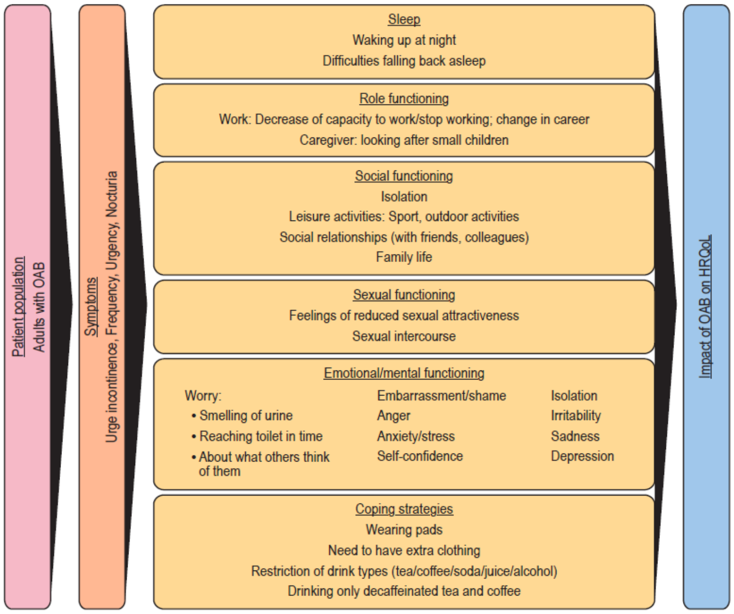

HRQoL: health-related quality of life; OAB: overactive bladder

## DISCUSSION

The present study was conducted to gain a better understanding of patients' perceptions of OAB and the effects of OAB symptoms on their HRQoL, particularly their self-image, and social and personal relationships. Qualitative data gathered from patient interviews revealed several important findings. Firstly, patient interviews indicated that OAB influenced the quality of life of patients in our study and adversely affected a broad range of functions, including role functioning, social functioning, emotional and mental functioning and sleep. Secondly, our study suggests that OAB-wet has a greater impact on social and emotional/mental functioning than OAB-dry.

A key element in our study was the effect of urinary urgency on psychosocial functioning, a finding which is consistent with the observations of Nicolson et al.[17] Eighty percent of our patient sample reported feeling embarrassment or shame as a result of their condition, usually because of how often they needed to go to the toilet or because of incontinence. Patients also reported a range of other emotions caused by OAB including worry, annoyance, stress or anxiety and anger. Some patients waited for up to 17 years before mentioning their condition to their GP because of shame and the perception that nothing could be done about it. Some of this shame was driven by the fear of smelling of urine. There is a social stigma associated with urination because urine has a strong and unpleasant smell, and people smelling of urine are generally seen as dirty and their incontinence signifies lack of self-control.[18]

Another key finding was the negative effect of OAB on sleep. Most patients had nocturia (93%) and all but one patient (97%) reported waking at night because of OAB. The run-on effects of broken sleep patterns were profound, resulting in tiredness, irritability, impaired daytime functioning and concentration. Similar observations have been made in other qualitative studies.[16,17] Some patients reported a decline in their work performance and general daily functioning as a result of fatigue. Others reported that they were forced to go to bed early to compensate for lack of sleep and had no social life as a result.

OAB symptoms also often limited day-to-day activities leading to loss of self-confidence, social withdrawal and isolation. Patients used the terms 'miserable' and 'ruined' to describe the dramatic impact of OAB on their daily functioning. Patients often preferred to stay at home rather than worry about using other peoples' toilets or looking for a public toilet. In the most extreme case, one patient reported that she no longer left the house because of fear of incontinence. These observations are consistent with a large European survey,[19] in which 76% of individuals stated that OAB symptoms had limited or interfered with their daily activities.

Patients commonly reported using non-medical coping strategies to manage their condition and to alleviate symptoms. The use of various coping strategies has been documented previously in adults with OAB.[20] The most common strategies, which were described by 50% to 60% of patients, were planning activities, restricting places visited or restricting fluid intake. Approximately one-fifth of patients reported wearing incontinence pads; the majority (5 out of 7) were OAB-wet patients. The use of coping strategies suggests that patients are trying to minimize the discomfort and embarrassment brought on by urgency or urge incontinence.

As part of the present study, we performed a literature search for qualitative studies investigating PRO related to OAB symptoms and management. From this search, we were able to identify only three published studies.[15–17] The findings of these studies were used to develop a preliminary conceptual framework for OAB. This was then extended and refined using the data gathered from our own patient concept elicitation interviews (Figure 1), as recommended in good research practice guidelines.[10] This framework clearly demonstrates the far-reaching effects of the symptoms of OAB on all areas of patient functioning and the need for coping strategies.

There are many existing measures for assessing symptoms and HRQoL in patients with OAB;[9] however, only a few consider the psychosocial aspects of the condition.[8,21–23] OAB-q, a widely used and validated OAB instrument, consists of an 8-item Symptom Bother Scale and a 25-item scale assessing HRQoL domains (i.e. coping, concern, sleep and social interaction).[8,9] It was specifically designed to assess HRQoL in both continent (dry) and incontinent (wet) patients with OAB,[9] and was found to discriminate between both of these patient subgroups.[8] Overall, there was a marked overlap between the questions in the OAB-q and the concepts identified in our study, indicating that our conceptual framework supports the OAB-q. There were, however, some points of difference between OAB-q and our conceptual framework. We identified more concepts relating to emotional/mental functioning (e.g. depression, irritability) and coping (e.g. wearing pads, restriction of fluids) than are covered by the OAB-q. Further, some of our patients indicated that the ability to work had been impaired by OAB, but OAB-q has no questions of this nature. The work-related effects of OAB on HRQoL could potentially be identified from items in the EuroQol-5 Dimension questionnaire (e.g. usual activities or anxiety/depression)[24] or the Work Productivity and Activity Impairment questionnaire (WPAI).[25] These findings indicate that a disease-specific questionnaire plus a generic and/or other questionnaires (e.g. WPAI) may provide a more complete picture of HRQoL in patients with OAB.

It should be noted that our study had several limitations. We developed an interview guide to avoid undue influence of the interviewer in patient interviews. The interview guide was developed with input from urologists, rather than OAB patients or existing literature on OAB, which may have limited its relevance to patients. The study sample was relatively small, although saturation was reached according to the study methods meaning that it was unlikely that adding new participants would add new information. There may have been some selection bias in our study sample, e.g. patients who did not seek medical attention or who had depression were under-represented. The inclusion criteria also required that patients were at least "somewhat" bothered by symptoms of urgency or urge incontinence, which may limit the generalizability of our findings to those with other levels of symptom bother. As most patients (90%) did not offer information on sexual function, this area was not well captured in our study. This is perhaps surprising given OAB is reported to impair sexual activity and increase dysfunction.[15,26] However, it is possible that the patients in our study felt uncomfortable discussing this topic or it was a minor issue because of limited sexual activity among older patients.

## CONCLUSION

This qualitative study indicates that OAB influences the quality of life of patients and has a negative effect on a broad range of functions, including role functioning, social functioning, emotional and mental functioning and sleep. The data also indicate that OAB has a greater impact on some HRQoL concepts in OAB-wet patients compared with OAB-dry patients. Self-management and coping strategies are adopted by patients to help manage their condition. The conceptual framework emerging from this study supports the OAB-q instrument, but identifies that work-related effects, which are relevant for OAB patients, are missing from currently available measures.

## Figures and Tables

**Figure:** Supplementary Content

